# Evidence of the drying technique’s impact on the biomass quality of *Tetraselmis subcordiformis* (Chlorophyceae)

**DOI:** 10.1186/s13068-023-02335-x

**Published:** 2023-05-20

**Authors:** Hareb Aljabri, Maroua Cherif, Simil Amir Siddiqui, Touria Bounnit, Imen Saadaoui

**Affiliations:** 1 Algal Technologies Program, Centre for Sustainable Development, College of Arts and Sciences, Qatar University, P.O. Box 2713, Doha, Qatar; 2 Department of Biological and Environmental Sciences, College of Arts and Sciences, Qatar University, Doha, Qatar

**Keywords:** DHA, Drying technique, Biomass quality, PUFA, *Tetraselmis subcordiformis*

## Abstract

Rapid drying, cost-effective and safe, will increase the viability of using microalgae for several bio-industrial applications. In this study, five different drying techniques of microalgal biomass were investigated. These include freeze drying, oven drying, air drying, sun drying, and microwave drying. Morphology, metabolite content, FAME profiling, chlorophyll content, total organic carbon, and total nitrogen were analyzed. Results showed that the freeze-drying technique preserves the highest amounts of chlorophyll, proteins, and lipids. Oven drying underperformed as it retained the lowest amount of chlorophyll, protein, and lipid content. More importantly, FAME profiling results showed that air drying was the best technique in maintaining the highest amount of polyunsaturated fatty acids and more specifically docosahexaenoic acid (DHA). Furthermore, this process requires the least capital and energy needs. The findings from this study confirmed that the drying technique affects the microalga biomass quality.

**Graphical Abstract:**

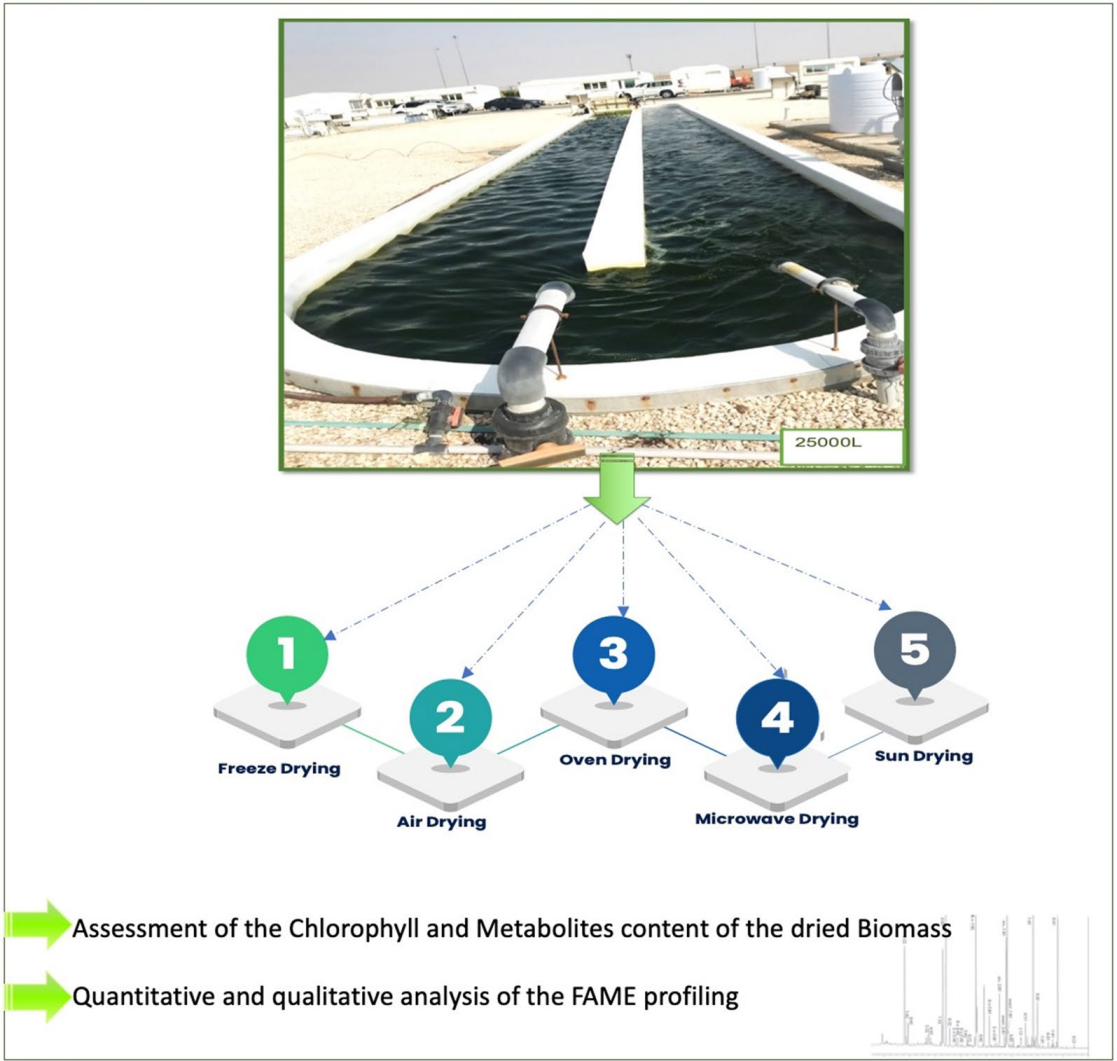

## Introduction

Microalgae have drawn the attention of many researchers in recent times as a reliable and renewable source of energy [1]. They have also been introduced as the third-generation biofuel and can yield up to 30 times more energy per area unit compared to first- and second-generation biofuels [2]. Furthermore, they have a higher growth rate, carbon fixing ability and elevated lipid production compared to terrestrial plants [3]. The major components of interest harvested from microalgae include proteins, lipids, carbohydrates and minor concentrations of vitamins, pigments, and sterols [4].

Microalgae can be cultivated at a large scale in photobioreactors or raceway ponds [5]. The cultivation of strain of interest is followed by harvesting the final biomass through a series of steps including biomass separation, screening, thickening, dewatering, and drying and lastly biorefinery for extracting the products of interest [3]. It is crucial to optimize these steps for the efficient production of high-quality algal biomass.

Dewatering and drying are vital elements in downstream extraction; therefore, it is important to consider the different techniques and their impacts on the cost and energy and most importantly the quality of biomass, high value products, and metabolites. Dewatering microalgae ensures effective processing downstream by removing majority of the water which lowers the cost and energy needed for the following drying steps [6], although dewatering requires approximately 20–40% of the energy requirements of the whole microalgal harvesting process. In addition, contamination risks need to be eliminated in case of production for human or animal consumption [7].

The drying process is also as important as the dewatering of microalgae. It is considered a crucial step as the algae slurry achieved from the upstream harvesting processes can be fragile. According to Patil et al. [8], drying process requires the most energy, accounting for over 80% of the total cost of manufacturing algal-based products such as biodiesel. As algae can be susceptible to microbial contamination, mechanical damage and adversary storing conditions, which may lower the quality of biomass, it is important to dry algae efficiently for optimal storage [9]. There are many ways of drying algae such as conventional sun drying, hot air drying, freeze drying, microwave drying, oven drying and spray drying [10].

Conventional methods such as sun drying and oven drying are usually pursued as these methods do not require high energy and capital input. But these methods may not be preferred for reasons such as susceptibility to contamination from outside sources such as birds, insects, and microorganisms in the case of sun drying [11]. Furthermore, this method relies heavily on the weather conditions and may not be feasible for areas with high rains and low sunlight. Another reason is the degradation of pigments such as chlorophyll due to direct solar radiation [12]. In addition, oven drying can negatively impact the heat-labile metabolites and bioactive compounds [13].

On the other hand, processes such as freeze drying and spray drying have become more common for drying the algae biomass. Freeze drying can be one of the safest forms of drying in terms of retaining important byproducts which may be lost otherwise, while spray-drying process can be time efficient and produce high-value products [14]. The disadvantages of these methods include high operational and maintenance costs. In addition, the spray-drying method includes high-pressure mechanisms, which may rupture cells and degrade high value-added components such as pigments [15].

Finally, the choice of drying depends on the capital and energy sources available and on the importance of byproducts that need to be successfully attained from the algae harvest [16]. Even though downstream processes such as dewatering and drying can be vital in the extraction of valuable high-quality algal biofuels and feedstocks for food supplements or animal feed, there is a lack of research outlining the importance of these processes [17].

This study aims to investigate the effect of five different drying techniques on the nutritional quality of the microalgae biomass. For that purpose, an assessment of the chlorophyll, proteins, lipids, and FAME content was performed to select the most suitable drying technique leading to maintaining high-quality biomass that can be used ultimately for animal-feed production; therefore, it will enhance the viability of the large-scale biomass production under arid climate.

## Materials and methods

### Microalgae cultivation

A single colony of the local marine strain *Tetraselmis subcordiformis* (*T. subcordiformis*) QUCCCM50 was used to inoculate 5 mL f/2 growth medium. The culture was incubated for 7 days in an illuminated shaker (Innova 44R, New Brunswick Scientific, USA) under the agitation of 150 rpm, an illumination of 100 μmol s^−1^ m^−1^ with a light–dark cycle of 12:12 h and a temperature of 30 °C, corresponding to the annual average temperature in Qatar. Subsequently, the culture was scaled up to 50 mL, then 200 mL, and then 1 L. All these cultures were incubated under the same previously described conditions. The 1 L culture was used to inoculate 5 L f/2 growth medium prior to being incubated under air bubbling, the light of 400 μmol s^−1^ m^−1^ and room temperature. Ultimately, the 5 L was scaled up to 20 L and incubated under the latter conditions of light, temperature, and air bubbling. The cultures were performed in triplicate.

### Drying techniques of the microalgae biomass

Five different techniques were tested such as (i) freeze drying (FD); (ii) air drying (AD); (iii) sun drying (SD); (iv) oven drying (OD) and (v) microwave drying (MD). For that purpose, 1 L sample of *T. subcordiformis* culture was collected from each tank on the same day for each technique to be tested. The drying was performed in triplicate using 1 L from each tank of 20 L (1 L sample per tank, and three replicates per drying technique). After harvesting using a cold centrifuge (SL16R, Thermo Fisher), the wet biomass was spread into a glass plate prior to being dried via the following techniques:

- Freeze drying (FD): After an ON incubation at − 80 °C, the biomass was transferred into a freeze dryer (Labconco, 7754067) for a duration of 48 h,
- Sun drying (SD): wet biomass was incubated directly under the sun for 48 h in a location that allows the maximum of illumination hours,
- Air drying (AD): biomass was covered with 4 layers of aluminum and incubated at room temperature for 48 h,
- Microwaves drying (MD): wet biomass was incubated in a microwave (Panasonic NN-CD9975) for a duration of 2 h with a successive incubation of 15 min until completely dry.
- Oven drying (OD): the biomass was incubated for 48 h in an oven (Binder EP-53) under 90 °C.

Treatments were performed in parallel and stopped after confirming a stable weight of the dry biomass.

#### Moisture content determination

1 L of wet biomass was used for each drying technique and weight difference before and after drying technique was recorded. This difference was used to calculate the moisture content % after the drying process for each technique.

### Morphological characterization of *T. subcordiformis* under the different drying techniques

The morphology of the dried biomass of *T. subcordiformis* was investigated via light microscopy using light microscopy (40 ×, Primo Star HAL Microscope, full Köhler, stage drive R, FOV 20, Carl Zeiss, Germany) and Scanning electron microscopy (SEM). For the SEM characterization, the dried algal cells issued from the different drying techniques were dehydrated in gradually increasing ethanol concentrations (up to 96% ethanol), and then transferred in formaldehyde dimethyl acetal for 24 and 2 h, critical point dried with CO2, sputter coated with palladium/gold and examined with a (Nova™ Nano SEM 50 Series).

### Metabolite’s characterization

The total lipids were extracted from the dried algae biomass issued from the six previously described drying methods using a modified method of Folch et al. [18]. After extraction and drying, the total amount of lipid was determined gravimetrically and the lipid content (%) was determined using the following equations according to Arora et al. [19]:

$${\text{Lipid content}}\,\left( \% \right) = {\text{Total lipids}}\,\left( g \right)/{\text{Dry biomass}}\,\left( g \right)$$

The total proteins content (% per g dry biomass) was assessed as per Saadaoui et al. [20]. 100 mg of dry microalgae biomass was subjected for total proteins extraction using a Sigma kit (Plant Total Protein Extraction PE0230, USA). Then, the total proteins were quantified using the Bradford assay [21].

For chlorophyll extraction and quantification, 50 mg of the dried *T. subcordiformis* issued from the 6 different methods tested were suspended in 1 mL of methanol 90% and kept at 60 °C water bath until the biomass was colorless for chlorophyll extraction. Then, the mixture was centrifuged at 1500 × g for 5 min after reaching room temperature. The chlorophyll concentration was calculated using the equations described by Porra et al. [22] after reading the extract absorption at different wavelengths (650 and 665 nm) using a spectrophotometer (Jenway, 6305, UK).

### FAME profile determination

Fatty acid methyl esters (FAMEs) were extracted using a one-step transesterification method [20]. For that purpose, 50 mg of dried biomass issued from the different drying techniques was added to an adequate volume of sulfuric acid (95%) and methanol (H2SO4:CH3OH, 1:10). Then, after 10 min of sonication (Branscon 1510, Mexico), the mixture was incubated for 2 h at 80 °C, and then transferred to a centrifuge tube containing distilled water and a mixture of hexane: chloroform (4:1). Finally, the FAME fraction was stabilized by the addition of BHT, followed by filtration, prior to GC-FID (Shimadzu, Japan) analysis.

### Total organic carbon and total nitrogen of *T. subcordiformis*

Sample preparation included grinding the dried sample using a mortar and pestle. For determining the total organic carbon (TOC), 0.5 mg of finely ground sample was placed into silver capsules. Acid (HCl) was added to the sample to eliminate all inorganic carbon in the form of carbon dioxide. The capsules were closed and fed into the autosampler of the FLASH 2000 NC Soil Analyzer (Thermo Scientific™, United States). For determination of Total Nitrogen (TN), 0.5 mg of dried finely ground samples was placed into tin capsules. The capsule was directly fed into the autosampler of the FLASH 2000 CHNS/O Organic Elemental Analyzer (Thermo Scientific™, United States).

### Statistical analysis

All the drying techniques as well as the metabolic characterization were performed in triplicate. The reported values are the means of three independent samples while the error bars represent standard error. One-way ANOVA was used to determine significance difference (*α* = 0.05) between means. Freeze drying was used as reference in this study, to compare if there was a significant difference in experimental results for the techniques as it is the most conventionally used drying method.

## Results

### Characterization of the biomass and the cell morphology of *T. subcordiformis* after applying the different drying techniques

**Fig. 1:**
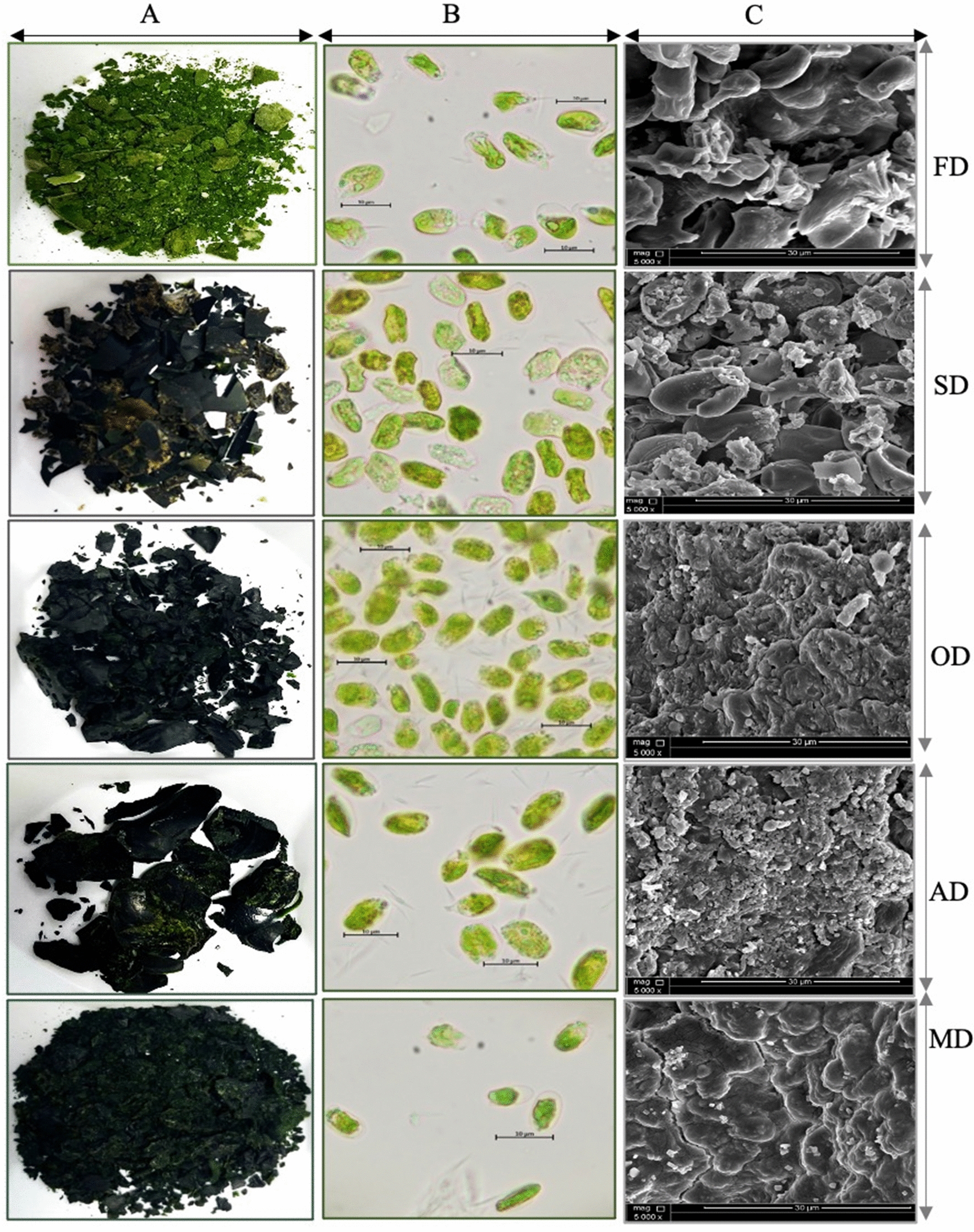
Biomass and cell morphology after different drying techniques. **A**: Biomass characterization and **B**: cell morphology under light microscopy with a magnification of 100X. **C**: Scanning electron microscopy HV: 10.00 kV, WD: 5.0 mm and magnification: 5000X. FD: freeze drying; MD: microwave drying; AD: air drying; SD: sun drying; OD: oven drying

The assessment of *T. subcordiformis* biomass quality generated from different techniques evidenced different colors and textures. The biomass issued from freeze drying was light green like the wet biomass, while the four other techniques led to biomass with a dark green color. We also noticed that the biomass issued from air drying, sun drying and oven drying led to very dry and hard biomass. Moreover, sun drying led to the appearance of a specific orange color that might correspond to carotenoids production (Fig. 1a).

Furthermore, we noticed that the *T. subcordiformis* cell morphology was specifically impacted by the drying technique applied. Results proved that freeze-dried cells presented similar morphology to the wet cells with a length of 10 μm. However, cells subjected to microwave and oven drying showed smaller sized cells with almost no internal vesicles, while for sun-dried biomass irregular shape with a small size was observed with the appearance of orange vesicles, although air-dried cells presented regular size and shape but contained fewer vesicles (Fig. 1b). The scanning electron microscopy also evidenced differences (Fig. 1c). Both freeze- and sun-dried cells were separated from each other. The freeze-dried cells were intact; however, the sun-dried cells were broken, while the other three techniques led to cells piling together.

### Moisture and chlorophyll content of the biomass after different drying techniques

The moisture content for the various drying techniques was not significantly different. The highest moisture content was recorded for microwave drying while the lowest was for sun drying. All drying techniques were carried out for 48 h which ensured very low moisture content, allowing for easier biomass analysis. Microwave drying was conducted for 2 h only hence, the higher moisture content (Table 1).

**Table 1:** Moisture content of *T. subcordiformis* biomass after drying using different techniques, experiment (*p* < 0.05)

Drying technique	Average moisture content (%)
Freeze drying	0.910 ± 0.0021
Sun drying	0.907 ± 0.0042
Oven drying	0.908 ± 0.0023
Air drying	0.908 ± 0.0026
Microwave drying	0.914 ± 0.0078

**Fig. 2:**
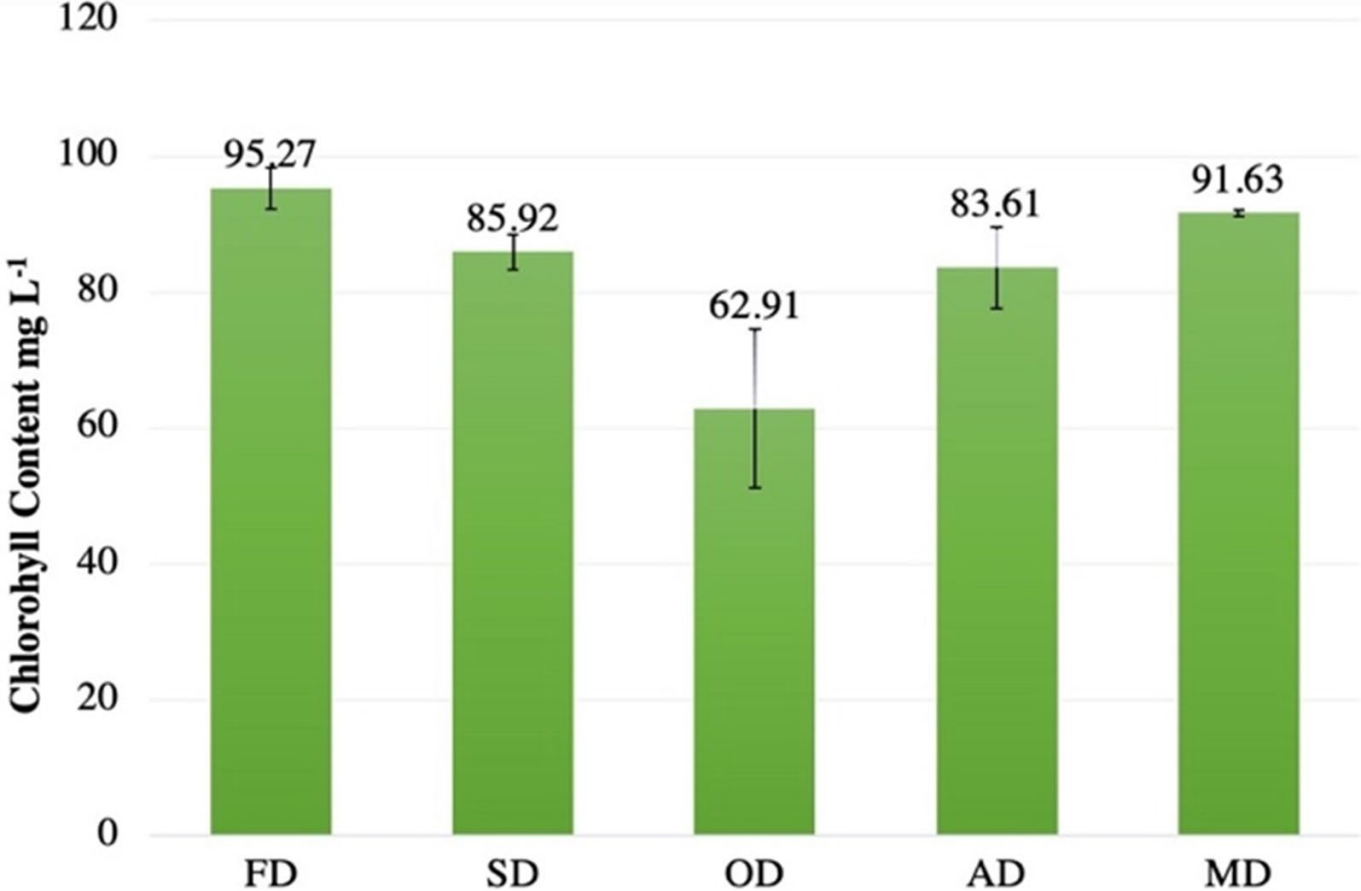
Chlorophyll content of the microalgae biomass after different drying techniques. FD, freeze drying; SD, sun drying; OD, oven drying; AD, air drying; MD, microwave drying. Error bars showcase the standard deviation of three replicates for each drying process

Results proved the presence of significant difference between the five different techniques in terms of chlorophyll content (*p* < 0.05). The highest chlorophyll amount was observed in the case of the freeze-dried biomass with 95.27 ± 3.01 mg L^−1^ followed by the microwave drying (91.63 ± 0.47 mg L^−1^, *p* = 0.05). The sun and air drying led to a similar chlorophyll amount of 85 mg L^−1^, *p* < 0.05. The lowest amount of chlorophyll was detected in the case of oven drying (62.6 ± 7.9 mg L^−1^, *p* < 0.05) with a decrease of 34% compared to the freeze-dried biomass (Fig. 2).

### Assessment of the metabolites content of the biomass after different drying techniques

**Fig. 3:**
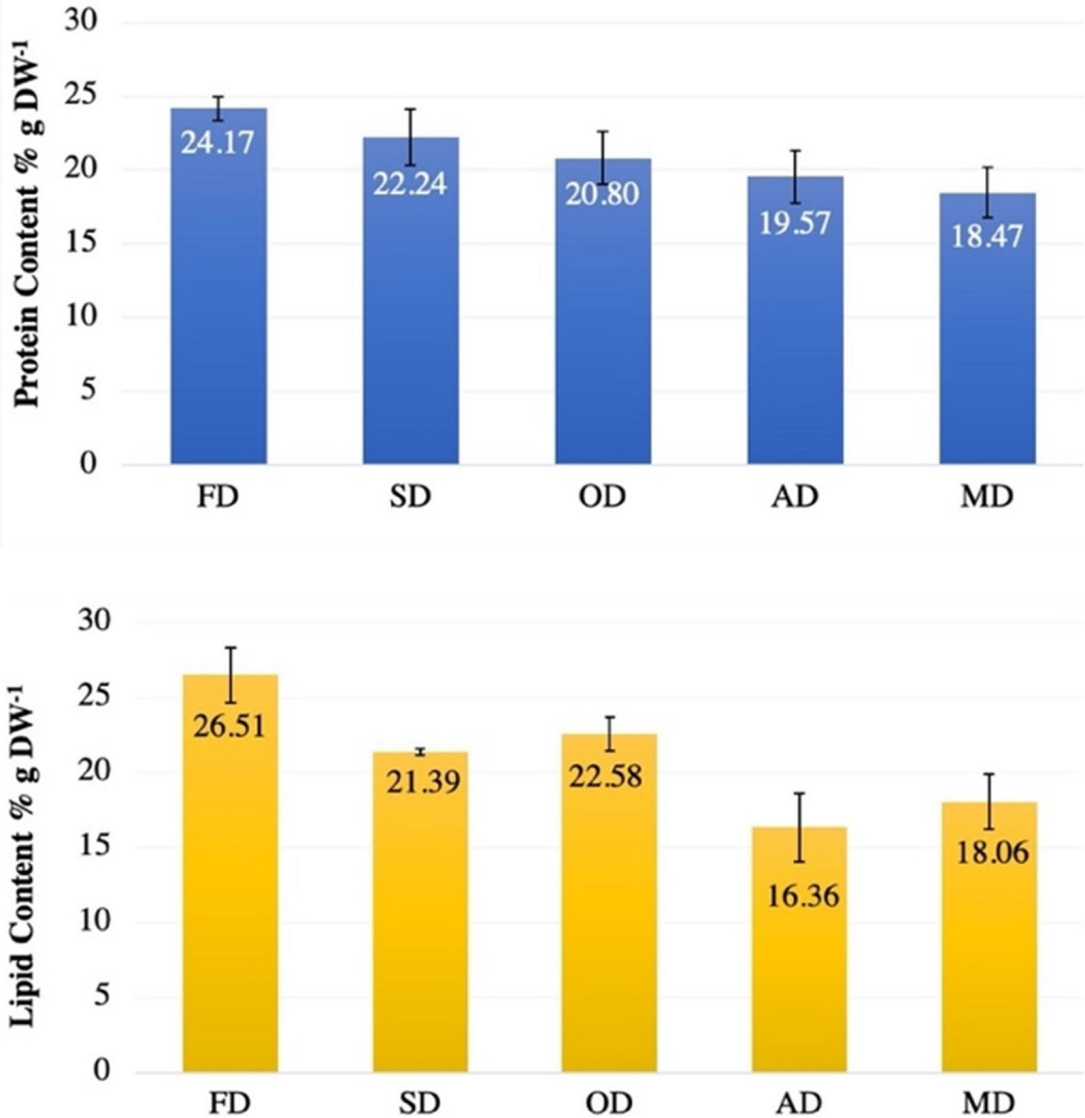
Metabolite content of the *T. subcordiformis *biomass dried under the different drying techniques. **A** Content and **B** lipid content. FD, freeze drying; MD, microwave drying; SD, sun drying; OD, oven drying; AD, air drying. Error bars showcase the standard deviation of three replicates for each drying process

A significant difference was observed between protein contents of the microalgal biomass obtained after applying the different drying techniques when compared to freeze-drying technique (*p* < 0.05) except for protein content of the sun-drying method (*p* = 0.124). The highest protein content was observed in the case of freeze drying with 24.17 ± 0.78% dry wt^−1^, followed by sun drying (22.24 ± 1.86% dry wt^−1^) then oven drying (20.8 ± 1.86% dry wt^−1^). The least amount was observed in the case of air and microwaves drying showing a similar amount of ~ 19% dry wt^−1^ (Fig. 3a).

Similarly, to the protein content, a significant difference was observed between the different tested techniques in terms of lipid content (*p* < 0.05) and the highest amount was observed in the case of freeze-dried biomass 26.51 ± 2.26% dry wt^−1^ followed by biomass issued from sun and oven drying with a similar amount of ~ 21% dry wt^−1^. The least amount was also observed in the case of air and microwaves drying showing a lipid content of ~ 17% dry wt^−1^ (Fig. 3b).

### Assessment of the FAME profiling of *T. subcordiformis* after the different drying techniques

Results proved that the FAME composition was heavily affected by the drying technique (Table 2). The highest total amount of FAME was observed in the case of freeze drying (522.65 ppm) followed by air drying (159.44 ppm), and then microwaves drying (134.51 ppm). Oven drying and sun drying led to a similar amount of FAME ~ 80 ppm. Regarding PUFAs amount, we noticed that the air drying showed the highest amount of 80.92 ppm, followed by the freeze drying (68.1 ppm), then microwave drying (50.72 ppm), and then oven drying (39.33 ppm). However, the sun drying led to the lowest PUFAS amount of 9.23 ppm. Moreover, results evidenced that ALA (C18:3) was observed under the five different techniques; however, significantly different amounts were observed. Freeze-dried biomass showed the highest ALA amount with 44.09 ± 4.7 ppm followed by air-dried biomass (25.25 ± 1.25 ppm), then microwave drying (16.02 ± 2.9 ppm). Sun drying and oven drying showed similar and very low ALA amounts of 5 ppm.

More importantly, we noticed that the DHA (C22:6) was present only in the case of air drying, microwave drying, and the highest amount was observed in the case of air drying (9.89 ± 3.45 ppm). Finally, we noticed that sun drying and oven drying resulted in the total absence of the DHA.

### Total carbon and nitrogen of the microalgae biomass after the different drying techniques

Results proved the absence of significant difference in terms of nitrogen and hydrogen amounts of the microalgae biomass obtained from the drying techniques (Table 3). However, slight differences were observed in the case of total organic carbon. Freeze drying led to the highest TOC of 35.18% weight basis and microwave drying led to the lowest (30.70 ± 0.033% weight basis). The four other techniques led a very similar TOC of ~ 33% weight basis (Table 3).

**Table 2:** FAME profiling of the *T. subcordiformis* biomass obtained after the different drying techniques

FAME (ppm)	Freeze drying	Sun drying	Oven drying	Air drying	Microwaves drying
C16:0	481 ± 46.56	32.68 ± 1.6	30.32 ± 5.16	52.92 ± 1.34	58.81 ± 7.7
C16:1	17.56 ± 1.41	–	–	–	3.09 ± 0.9
C18:0	30.71 ± 3.00	2.86 ± 0.1	5.08 ± 0.82	4.80 ± 0.47	5.74 ± 0.7
C18:1 (Oleate)	14.58 ± 1.14	3.48 ± 0.9	5.85 ± 3.51	–	–
C18:1 Vaccenate	15.62 ± 3.21	1.36 ± 0	1.31 ± 0.27	2.46 ± 0.16	2.36 ± 0.3
C18:2	9.99 ± 2.21	0.54 ± 0.1	0.63 ± 0.38	4.12 ± 0.3	4.50 ± 0.5
C18:3 (ALA)	44.09 ± 4.7	4.61 ± 0.2	6.41 ± 1.31	25.25 ± 1.25	16.02 ± 2.9
C20:0 Arachidate	4.32 ± 0.82	7.09 ± 0.4	7.71 ± 1.31	7.93 ± 0.24	6.65 ± 1.2
C20:1	8.13 ± 1.48	–	–	4.24 ± 0.15	4.19 ± 0.5
C20:2	14.02 ± 4.74	4.08 ± 0.2	5.15 ± 0.87	18.61 ± 0.68	12.49 ± 1.9
C20:4	–	–	27.15 ± 4.59	23.05 ± 1.79	12.99 ± 2.5
C22:6 (DHA)	–	–	–	9.89 ± 3.45	4.72 ± 1
C24:0	6.62 ± 0.92	23.26 ± 1.6	–	6.17 ± 1.47	2.94 ± 0.2
Total FAME	522.65	79.96	89.59	159.44	134.51
SFA	522.65	65.89	43.11	71.82	74.14
MUFA	55.89	84.80	96.75	166.14	144.17
PUFA	68.1	9.23	39.33	80.92	50.72

**Table 3:** Microalgae biomass properties after the different drying techniques

	N % dry weight basis	TOC % dry weight basis	H % dry weight basis
Freeze drying	4.62 ± 0.04	35.18 ± 0.06	6.21 ± 0.06
Sun drying	5.06 ± 0.03	32.93 ± 0.5	5.97 ± 0.06
Air drying	5.06 ± 0.12	32.84 ± 0.15	6.12 ± 0.06
Microwaves drying	4.48 ± 0.01	30.70 ± 0.033	5.14 ± 0.08
Oven drying	5.36 ± 0.1	34.04 ± 0.15	5.85 ± 0.14

N, nitrogen, H, hydrogen, TOC, total organic carbon

## Discussion

This study investigates the various drying techniques to determine the best possible method for the preservation of strain and biomass quality. Extensive analyses were conducted to view all the possible changes the various drying techniques can produce. Produced biomass and morphology assessment showed that the most suitable method is freeze drying as it preserves the cell size of the microalgae, which was like the cell size in wet biomass (Fig. 1b). This study’s findings align with Min et al. [23] study, which states that freeze drying does not harm the strains cell wall, hence producing a high-value product. On the other hand, air drying, sun drying and oven-drying technique which are the easiest to perform produced extremely dry and stiff biomass. Scanning electron microscopy confirmed this constatation as it revealed a surface with cells attached to each other in the form of a membrane. The morphology of air-dried cells showed less vesicle formation, and sun-dried cells showed irregular cell shape with orange vesicle formation. These changes may be due to internal changes in the cell, for example, vesicle loss from air-dried samples may be the reason for low lipid content in the biomass obtained and sun drying may have led to the deterioration of green pigments and production of carotenoids, as response to the light stress, leading to change in color, also noted in Hosseinizand et al. [24] study. The presence of carotenoids was confirmed by spectral scanning showing high peak of absorbance at 420 nm. These pigments were released outside the cells and led to the orange color of the sun-dried biomass. This was confirmed by scanning electron microscopy proving the sun-dried cells presented a broken cell membrane (Fig. 1c).

The highest chlorophyll content was visible in freeze-dried samples, this was possible as this method is safe and preserves the quality of cells, ensuring the viability of pigments in the biomass and no degradation takes place. These results also align with Shekarabi et al. [25] study, which compared chlorophyll content in multiple drying techniques. The highest chlorophyll content was noted for freeze drying (1.507 μg/m). The second-best method for chlorophyll pigment preservation in cells was microwave drying. This may be because it is the least time-consuming method with only 2 h of drying, which may have ensured that the pigment is not lost. De Farias Neves et al. [9] suggest that chlorophyll concentration can decrease significantly after 40 °C and that temperature for dying should be optimized beforehand, while the lowest chlorophyll content was found to be, in biomass obtained from oven drying, with 34% less chlorophyll compared to the freeze-drying method. All drying methods other than freeze and air drying, utilize a heat source, which explains the decrease in chlorophyll content for most heat using methods, except for microwave, and this may be due to thermal degradation of the pigments [24, 26]. In addition, a study on Spirulina strain showed adverse effects on the cell by products and on pigments at temperatures exceeding 45 °C [27].

The same trend was observed for both lipid and protein content, the maximum value was retrieved from biomass that was freeze-dried. In a study by Zhang et al. [28], the lipid content of freeze-dried Scenedesmus was noted to be 33% compared to 26.5% in the current study. The difference in lipid content may be accounted by the methodology adopted for freeze drying, where the temperature was set to − 50 °C over 72 h. In another study, freeze and air drying were compared for future commercialization prospects. Two microalgae strain *Chaetoceros *sp*.* and *Phaeodactylum tricornutum* were used and lipids were profiled. It was found that air drying led to the loss of almost 70% of the lipids compared to freeze drying [29]. The second most effective method for metabolite preservation was noted to be oven drying, followed by sun drying and the lowest amounts were observed in the case of microwaves and air drying that also present similar results. On the contrary, a study comparing oven-drying and microwave-drying technique concluded that lipid yield (28.35%) was higher in microwave drying at lower power (540 W) compared to oven drying (19.14%). This is due to uniform heating and less drying time in microwaves compared to oven drying where higher temperatures and time is required [30]. Results of this study aligned with the findings of Desmorieux and Hernandez [31] who analyzed five different drying techniques on spirulina cyanobacterium and proved that freeze drying retained the highest amount of sugar and protein. Furthermore our results of higher protein production in high temperature oven drying aligns with the research of Nelson [32], but it was suggested that this may be an anomaly and may be due to increased signal through higher cell digestibility in high heat.

FAME profiling analysis showed that the provision and absence of different fatty acids depended on the drying method used. For example, even though the total FAME was highest for freeze-dried biomass, it retained the lowest amount of MUFAs. Most of the total FAME from freeze-dried sample can be attributed to SFAs. Furthermore, highest concentration of ALA was noted but DHAs were completely absent in the freeze-dried biomass. In a study by Nelson [32], it was noted that drying using heat above 60 °C prevents the conversion of triglycerides to free fatty acids, due to lipase deactivation. This may be the reason for the lowest concentration of FAME for all drying methods which use heat, especially sun drying and oven-drying methods. On the other hand, an unexpected finding was the highest concentration of MUFAs and PUFAs found in air-dried biomass, also with the highest amount of DHAs. This may be as the sample is dried naturally under room temperature conditions, these fatty acids are preserved and may be heat sensitive, and hence the other drying techniques may have reduced them. Following air drying, microwave showed higher concentration of FAME, as suggested by Nelson [32] that shorter heating time at high heat is better than heating for longer time which is the case in oven and sun drying. Accordingly, air drying is the most suitable drying technique to produce algae biomass enriched with omega-3 fatty acids.

Overall, through all the analyses conducted, the best suitor for preservation of biomass quality, freeze drying takes the lead, although it does have some disadvantages, which can be significant when deciding which drying method to use. For example, freeze drying requires 45.75 kWh/kg of energy, while comparatively solar and microwave drying require only 0.01–0.1 and 26.2–34.9 kWh/kg, respectively [33]. Another disadvantage may be the large capital investment needed for large-scale algal product recovery [34], sun drying is an easy and energy-efficient method but requires a large area and can depend on the weather of site, making it unreliable. Furthermore, in our study, sun drying did not provide significant results. Furthermore, it was reported that cell destruction and transmutation can be avoided below a certain critical temperature. Figuring out this temperature can be vital for the strain being used as it will avoid loss of important byproducts produced by the cells [33].

## Conclusion

Drying techniques can heavily affect the *T. subcordiformis* metabolite composition and more specifically the pigment, lipid content and the FAME profile. Furthermore, this study provides a list of analyses which focus on the different byproducts of interest extracted from microalgal biomass such as pigments such as chlorophyll, proteins, lipids, and fatty acids more specifically PUFAs such as DHAs and ALAs. As noted in this study, depending on the product of interest, production scale and capital available, drying techniques should be considered accordingly [35]. However very few studies are reported on considering the different techniques and their effect on the byproduct yields with such holistic approach. Overall, air drying is the most suitable technique to be applied since for production of high value fatty acids such as PUFAs (ALA and DHA) and MUFAs while being low cost and environmentally friendly. Accordingly, this would enhance the viability of the large-scale production of high-quality nutritional supplement and other alternatives such as biodiesel as well.

## Data Availability

Not applicable.
